# Characteristics of total body and appendicular bone mineral content and density in Japanese collegiate Sumo wrestlers

**DOI:** 10.1038/s41598-022-15576-x

**Published:** 2022-07-12

**Authors:** Taishi Midorikawa, Suguru Torii, Megumi Ohta, Shizuo Sakamoto

**Affiliations:** 1 College of Health and Welfare, J.F. Oberlin University, 3758 Tokiwamachi, Machida, Tokyo, 194-0294 Japan; 2 Waseda Institute for Sport Sciences, Waseda University, 2-579-15 Mikajima, Tokorozawa, Saitama, 359-1192 Japan; 3 Faculty of Sport Sciences, Waseda University, 2-579-15 Mikajima, Tokorozawa, Saitama, 359-1192 Japan; 4 Faculty of Liberal Arts and Sciences, Chukyo University, 101-2 Yagoto Honmachi, Showa-ku, Nagoya-shi, Aichi, 466-8666 Japan; 5 Faculty of Sport Science, Surugadai University, 698 Azu, Hanno, Saitama, 357-8555 Japan

**Keywords:** Bone, Bone quality and biomechanics

## Abstract

The purpose of this study was to explore the characteristics of total body and appendicular bone mineral content (BMC, kg) and bone mineral density (BMD, g/cm^2^) in Japanese collegiate Sumo wrestlers. A total of 54 male college Sumo wrestlers were recruited for the study and assigned to two groups according to their body weight (middleweight, 85–115 kg, *n* = 23 and heavyweight, > 115 kg, *n* = 31). The BMC, BMD, fat mass, and lean soft tissue mass (LSTM) values were measured using dual-energy X-ray absorptiometry for the total body and appendicular regions. Heavyweight Sumo wrestlers had significantly higher body weight, fat mass, and LSTM compared to middleweight Sumo wrestlers. The mean total body and regional BMC values were significantly higher in heavyweight than in middleweight Sumo wrestlers. The total body and leg BMD was significantly higher in heavyweight than in middleweight Sumo wrestlers, and was significantly correlated with body weight, but not arm BMD. The present study indicates that BMC and BMD might not sharply elevate among even heavyweight athletes, although heavier Sumo wrestlers had a greater BMC and BMD.

## Introduction

Sumo wrestlers are required to weigh more than 100 kg to win a game. In general, although Sumo wrestlers could be considered obese, previous studies have reported that the mean value of the percentage body fat using underwater weighting method and dual-energy X-ray absorptiometry (DXA) was not too high (between 20 and 30%)^1,2^. Moreover, previous studies indicated that the average value of fat-free mass (FFM) in Sumo wrestlers was approximately 80 kg^2^ and the upper limit of FFM might approach 150 kg^3^. Thus, investigating the body composition of Sumo wrestlers might lead to the appropriate knowledge of adaptation ability in humans.

The FFM consists of various organ tissues. Skeletal muscle (SM) is the largest organ tissue^4^ and constitutes approximately 40% of the FFM in adults^5^. It was reported that the SM mass in college Sumo wrestlers with an FFM of 79 kg was approximately 37 kg and approximately one and a half times compared to that of untrained college students with an FFM of 53 kg (i.e. 25 kg)^6^. In addition, the left ventricular end-diastolic dimension of Japanese professional Sumo wrestlers frequently exceeds the traditionally accepted upper limit of normal for the general population^7^. Notably, the mass of liver and kidneys in Sumo wrestlers (2.4 kg and 0.5 kg, respectively) was also higher than that in controls (1.4 kg and 0.3 kg, respectively)^6^. These increased values might be one of the adaptations for daily exercise training and diet for Sumo wrestling.

Currently, there is limited information regarding the bone mineral content (BMC: kg) and bone mineral density (BMD: g/cm^2^) in Sumo wrestlers, although bone is the third-largest organ tissue of FFM components. Since it is well known that weight-bearing exercises may be more effective for increasing BMC and BMD^8^, it is hypothesized that heavier Sumo wrestlers could have a higher BMC and BMD. Therefore, the purpose of this study was to explore the characteristics of total body and appendicular BMC and BMD in Japanese collegiate Sumo wrestlers.

## Methods

### Participants

A total of 54 male college Sumo wrestlers, who belong to the top level of the division, were recruited for the study. More than half of the participants had a career spanning more than 10 years. Regular training (“Kei-ko”) does not target specific muscles but is more of a whole-body exercise for proper balance. None of the participants had a history of cardiovascular, endocrine, or orthopedic disorders, nor had they ever tested positive for anabolic steroids or taken any medication during the given time of measurement. This study followed the guidelines of the Declaration of Helsinki, and the Ethical Committees of Waseda University and the National Institute of Health and Nutrition approved all the procedures involving human participants/patients. Written informed consent was obtained from all the participants prior to testing.

### Anthropometry and DXA measurements

The body weight was measured to the nearest 0.1 kg by using a digital scale (DC-320, TANITA Co. Ltd), with the participants wearing only minimal clothing. The standing height was measured to the nearest 0.1 cm by using a stadiometer (YS-OA, AS ONE Co. Ltd). The body mass index (BMI) was calculated as the body weight in kilograms per square of standing height in meters (kg/m^2^). The percentage of body fat was measured using DXA (Delphi A-QDR, Hologic Inc., Bedford, MA, USA; Version 12.4:3 Auto Whole Body Fan Beam). The FFM was calculated from the body weight and the percentage of body fat.

The total and appendicular (arm and leg regions) BMC and BMD, fat mass, and lean soft tissue mass (LSTM) were also measured using DXA. All the participants fit into the scan area of DXA. The whole-body scan image was separated into discrete regions using DXA regional computer-generated default lines on the anterior planogram view with manual adjustments. The ‘Head and Neck’ region was defined as the area above the line connecting the bilateral acromions. The ‘Arm’ region was defined as the area distal to the line connecting the axilla and the glenohumeral joint space. The ‘Leg’ region was defined as the area distal to the line tangent to the ischium and the upper edge of the greater trochanter. The estimated coefficient of validation for DXA measurements from the test–retest analysis was determined to be < 1%. One anthropometry and set of DXA measurements were performed during a single-study visit to Waseda University either during the preseason or postseason.

### Statistical analysis

All the results are presented as means and standard deviations. A total of 54 participants were assigned to two groups according to a body weight of 115 kg, which is the criteria of the International Sumo federation between the middleweight (85–115 kg; *n* = 23) and heavyweight groups (> 115 kg; *n* = 31). The differences between the two groups were tested for significance using an unpaired t-test. A Pearson’s product-moment analysis was used to compare the relationship of body weight with BMC and between each BMD (total body, arms, and legs) and anthropometric parameters (body weight, fat mass, and LSTM) for all participants. Statistical analyses were performed using SPSS for Windows (IBM SPSS version 27.0 and 28.0; SPSS Inc., Chicago, IL, USA). The differences were considered as significant when the *P*-value was < 0.05.

## Results

**Figure 1:**
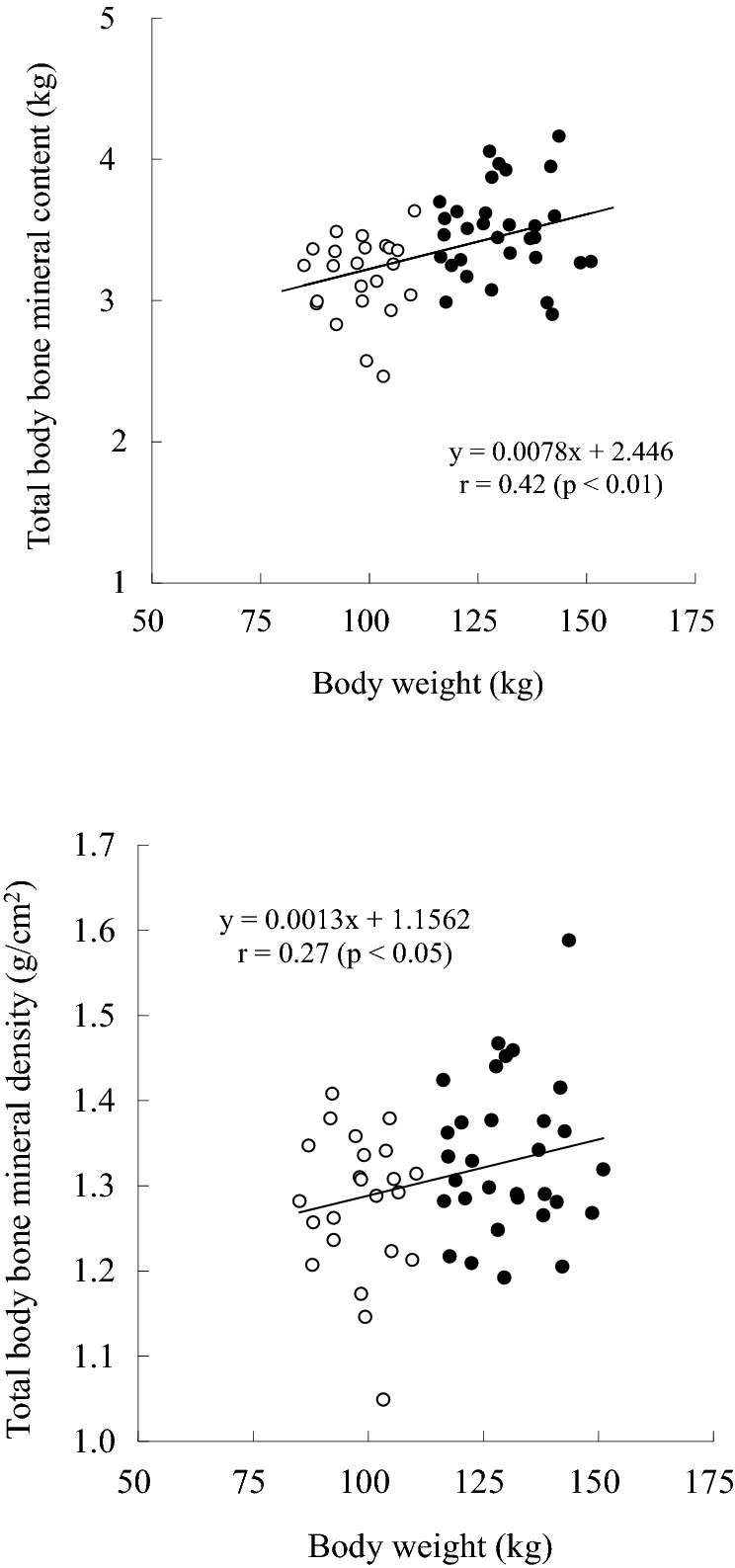
Relationships of body weight with total body bone mineral contents and density. ○: middleweight Sumo wrestlers, ●: heavyweight Sumo wrestlers.

Heavyweight Sumo wrestlers had significantly higher and greater standing height, body weight, BMI, body fat percentage, FFM, fat mass, and LSTM compared to middleweight Sumo wrestlers, as confirmed by DXA (Table 1). The mean total body and appendicular BMC were significantly higher in heavyweight than in middleweight Sumo wrestlers (Table 2). The percentages of each BMC to body weight were significantly lower in heavy weight Sumo wrestlers (Table 2). The body weight was directly proportional to the total body BMC in all the participants (Fig. 1). The total body BMD was significantly higher in heavyweight than in middleweight Sumo wrestlers and was significantly correlated with body weight but not with the fat mass and LSTM (Fig. 1, Tables 2 and 3). The BMD of the arm region was not different between both groups and was not associated with body weight, fat mass, and LSTM (Tables 2 and 3). The BMD of the legs was significantly higher in heavyweight than in middleweight Sumo wrestlers and was significantly correlated with the body weight and LSTM (Tables 2 and 3).

**Table 1:** Subject characteristics.

	Middle weight	Heavy weight
	*n* = 23	*n* = 31
Age (year)	20 ± 1	19 ± 1
Standing height (cm)	173.4 ± 5.8	177.0 ± 5.3*
Body weight (kg)	98.1 ± 7.4	130.4 ± 10.2**
BMI (kg/m^2^)	32.7 ± 2.9	41.8 ± 4.3**
Fat (%)	23.3 ± 3.4	31.2 ± 3.9**
Fat-free mass (kg)	75.1 ± 5.2	89.5 ± 5.6**
Fat mass by DXA (kg)	23.5 ± 4.4	41.5 ± 7.4**
Lean soft tissue mass by DXA (kg)	73.5 ± 5.2	87.2 ± 5.1**

Middle weight vs. Heavy weight: *p < 0.05, **p < 0.01.

**Table 2:** Total and regional bone mineral content and density.

	Middle weight	Heavy weight
	*n* = 23	*n* = 31
**Bone mineral content (kg)**		
Total body	3.17 ± 0.29	3.49 ± 0.32**
Arm	0.54 ± 0.06	0.59 ± 0.06**
Leg	1.16 ± 0.11	1.28 ± 0.13**
**Bone mineral content/body weight (%)**		
Total body	3.25 ± 0.38	2.69 ± 0.31**
Arm	0.55 ± 0.07	0.46 ± 0.06**
Leg	1.19 ± 0.14	0.98 ± 0.12**
**Bone mineral dencity (g/cm**^**2**^**)**		
Total body	1.28 ± 0.08	1.33 ± 0.09*
Arm	0.99 ± 0.07	1.03 ± 0.07
Leg	1.34 ± 0.09	1.41 ± 0.11*

Middle weight vs. Heavy weight: *p < 0.05, **p < 0.01.

**Table 3:** Correlation coefficients between bone mineral density and body composition parameters.

Sumo wrestlers (*n* = 54)	Body weight	Fat mass	LSTM
**Bone mineral density (g/cm**^**2**^**)**			
Total body	0.27*	0.24	0.23
Arm	0.25	0.23	0.23
Leg	0.29*	0.21	0.34 *

*p < 0.05. LSTM: lean soft tissue mass.

## Discussion

The present study found that heavyweight collegiate Sumo wrestlers could have approximately 10% higher total body BMC than middleweight Sumo wrestlers (Table 2). Moreover, the highest value of total body BMC was 4.16 kg with 143.6 kg body weight in the present study. According to a previous study in healthy Japanese adults, the mean and 95th percentiles of total body BMC were 2.5 kg and approximately 3.1 kg, respectively, for the age group of 20–29 years (140 men, mean body weight: 65.6 kg) using DXA (QDR-2000, Hologic Inc.)^9^, which is the same product used in the present study. Furthermore, according to the previous study on competitive athletes, the mean total body BMC is 4.32 kg in collegiate American football players (33 men, mean age: 22.3 years, mean bodyweight: 97.7 kg) using DXA (Hologic-WI, Hologic Inc.)^10^. In addition, when the present study obtained a regression equation between the body weight and total body BMC (Fig. 1), applied in the case of having 200 kg bodyweight, the total body BMC would be 4.01 kg. Based on the present and previous studies, it is indicated that the total body BMC is not easily increased in response to a higher body weight, and it was probably that the mean total body BMC in Sumo wrestlers with even 200 kg bodyweight might be approximately 4.0 kg, although it is not known exactly why American football players with a lighter body weight have a similar BMC to Sumo wrestlers.

It has been well documented that weight-bearing physical activity has a positive effect on the BMD^8,11^. In fact, an elite male soccer player (age 21.0 years, body weight: 77.8 kg, total body BMD: 1.23 g/cm^2^) has approximately 10% higher BMD than the general population (age: 24.3 years, body weight: 76.5 kg, total body BMD: 1.12 g/cm^2^) as confirmed by DXA (QDR-1000, Hologic Inc.)^12^. The above-mentioned previous study in healthy Japanese adults reported that the mean total body BMD was 1.12 g/cm^2^ for the age group of 20–29 years^9^. Compared to the BMD value of the previous study, middleweight (body weight: 98.1 kg, total body BMD: 1.28 g/cm^2^) and heavyweight (body weight: 130.4 kg, total body BMD: 1.33 g/cm^2^) Sumo wrestlers in the present study have been observed to have approximately 15% higher total body BMD (The highest value of total body BMD was 1.59 g/cm^2^ with 143.6 kg body weight). Moreover, there were significant correlations between the body weight and total body BMD in all participants (Fig. 1). As predicted from the regression analysis, in the case of 200 kg body weight, the calculated total BMD was 1.42 g/cm^2^. Although a recent study reported that a collegiate American football player with a mean body weight of 135.5 kg had mean and max total BMD of 1.63 and 2.11 g/cm, respectively, as confirmed by iDXA (Prodigy GE Healthcare Lunar)^13^, it was speculated that the mean total body BMD in Sumo wrestlers with even 200 kg bodyweight might be approximately 1.40 g/cm^2^ when using DXA manufactured by Hologic Inc.

In the present study, the BMD in the legs, but not in the arms, was observed to be significantly different between middle- and heavyweight Sumo wrestlers (Table 2). In addition, although the BMD in the arms was not correlated with body weight, fat mass, and LSTM, BMD in the legs was significantly related to body weight and LSTM (Table 3). These results suggested that BMD in the legs might receive a larger load and mechanical stress through daily living and exercise compared to BMD in the arms. In particular, it is speculated that the specific Sumo wrestling training, such as stamping with a heavy body mass, might be more effective for BMD in the legs.

Sumo wrestlers have large masses of SM, liver, kidneys, and heart^6,7^. Similarly, this study found that Sumo wrestlers had a higher BMC and BMD in comparison to that in previous studies, although the present study had a lack of controls. On the other hand, the present study indicates that BMC and BMD might not sharply elevate even among heavyweight athletes.

## Data Availability

The datasets used and/or analyzed during the current study are available from the corresponding author on reasonable request.

## Abbreviations

BMC: Bone mineral contents
BMD: Bone mineral density
BMI: Body mass index
DXA: Dual-energy X-ray absorptiometry
FFM: Fat-free mass
LSTM: Lean soft tissue mass
SM: Skeletal muscle
